# The stress history of soil bacteria under organic farming enhances the growth of wheat seedlings

**DOI:** 10.3389/fmicb.2024.1355158

**Published:** 2024-03-21

**Authors:** Muriel Ornik, Renata Salinas, Giona Antonacci, Martin Schädler, Hamed Azarbad

**Affiliations:** ^1^Department of Biology, Evolutionary Ecology of Plants, Philipps-University Marburg, Marburg, Germany; ^2^Department of Community Ecology, Helmholtz-Centre for Environmental Research – UFZ, Halle, Germany; ^3^iDiv – Centre for Integrative Biodiversity Research Halle-Leipzig-Jena, Leipzig, Germany

**Keywords:** microbes, organic, conventional, farming, climate changes, wheat

## Abstract

The effects of stress factors associated with climate change and agricultural management practices on microorganisms are often studied separately, and it remains to be determined how these factors impact the soil microbiome and, subsequently, plant growth characteristics. The aim of this study was to understand how the historical climate and agriculture to which soil microbes have been exposed can influence the growth characteristics of wheat seedlings and their associated bacterial communities. We collected soil from organic and conventional fields with different histories of climate conditions to extract microbes to inoculate wheat seeds under agar-based cultivation conditions. Within a growth period of 8 days, we monitored germination rates and time as well as seedling above-ground biomass and their associated bacterial communities. The results showed a positive interaction between conventional farming practices and an ambient climate for faster and higher germination rates. We demonstrate that soil microbial extracts from organic farming with experience of the future climate significantly enhanced above-ground biomass along with the diversity of bacterial communities associated with seedlings than other treatments. Such findings support the idea that organic agricultural practices not only mitigate the adverse effects of climate change but also promote the diversity of seedling-associated bacteria.

## Introduction

The frequency and severity of extreme weather events have been on the rise over the past few decades due to global climate changes and are projected to continue increasing in the future (IPCC, 2021). As a result, extreme drought conditions have led to significant reductions in crop yields, ranging from 50 to 70% (Eckstein et al., 2020). This decline in crop production poses a challenge to meet the growing demand for food production, which is expected to increase by at least 70% by 2050 (FAO, 2009). The intensive agricultural management, such as conventional farming, involves using high levels of chemicals and fertilizers to increase crop production but can cause land degradation and loss of biodiversity (Foley et al., 2005; Powers and Jetz, 2019). Organic farming methods do not apply synthetic fertilizers or pesticides, which can benefit the overall health of the ecosystem (Bonilla et al., 2012; Lori et al., 2017). Global climate change is likely to strengthen the negative impact of agricultural intensification on different ecosystem functions (Sünnemann et al., 2021). However, we have a limited understanding of how agricultural fields under conventional and organic farming practices can withstand extreme weather conditions and thus ensure food production.

Soil and plant-associated microbes can produce a variety of biological products that can make a significant contribution to crop stress resistance. Climate extremes and agricultural management can both have major impacts on microbes (Azarbad, 2022). The use of high levels of chemicals and mineral fertilizers in conventional farming has been shown to have a significant negative impact on soil characteristics (Dubey et al., 2019), which can adversely affect soil microbiomes (Lupatini et al., 2017). For example, conventional farming has been reported to negatively influence the composition and functioning of the soil microbial community, resulting in a decrease in microbial diversity and biomass (de Vries et al., 2012). On the other hand, agricultural lands that have been managed organically have been shown to have better soil abiotic components, including higher organic matter contents and soil water holding capacity (Lotter et al., 2003), which are essential for microbial growth and function. Accordingly, previous research reported an increase in soil microbial diversity (Hartmann et al., 2015) and abundance (Lori et al., 2017) under this agricultural practice.

In the context of plant microbiomes, beneficial microbes can support the host plants by aiding in nutrient absorption, suppressing pathogens, and protecting against challenging environmental conditions (Li et al., 2019; Azarbad, 2022), thus can directly impact agricultural productivity (Schmidt et al., 2019). By studying 40 different agricultural fields, Ricono et al. (2022) reported that organic farming increases the diversity and abundance of beneficial microbes in winter wheat root microbiomes compared to conventional farming. In another example, organic farming has been shown to support a more diverse, complex, and stable microbial network (based on metagenomics sequencing) than that of conventional farming in sugarcane phyllosphere microbiomes (Khoiri et al., 2021). However, as the impact of agricultural practices and the impacts of climate change on soil and plant microorganisms are often studied separately, it is not yet clear how these factors independently or interactively affect plant growth characteristics as well as diversity, and the composition of associated microorganisms.

The aim of this study was to understand how the historical climate and agriculture to which soil microbes have been exposed can influence the growth characteristics of wheat seedlings and their bacterial communities. To achieve that, we collected soil samples from the Global Change Experimental Facility (GCEF). In 2014, the GCEF was established as a field research station located at the Helmholtz-Centre for Environmental Research in Bad Lauchstädt, Saxony-Anhalt, Germany (Schädler et al., 2019). This research station is one of the largest field experiments to evaluate the combined impact of warming and altered precipitation patterns (as a future climate scenario), as well as different types of land management (such as conventional and organic farming) on various ecosystem processes. Previous studies conducted in this experimental field provided insights into the effects of predicted future climate change scenarios and different farming practices on soil invertebrate feeding activities and soil respiration rates (Siebert et al., 2020), soil microbiome in terms of community structure (phospholipid fatty acid analysis) and functional diversity (carbon substrate utilization) (Kostin et al., 2021). For example, Wahdan et al. (2021) showed that the organic agricultural practice significantly enhanced the total richness of arbuscular mycorrhizal fungi (AMF) associated with roots of wheat under future climate, thus enhancing plant nutrient availability. Therefore, this field experiment provides a unique opportunity to study the role of soil microbiome history in shaping the growth characteristics of wheat seedlings. Following soil collections from organic and conventional farming fields (farming history) under ambient and future climate conditions (climate history), we performed an experiment to determine which farming and climate conditions are better in terms of soil microbiomes to improve wheat seedling growth. Specifically, we want to address the following questions: can soil microbiomes selected by a different history of farming and climate affect the growth traits of wheat seedlings? and to what extent does the history of soil microbiomes shape seedlings’ bacterial communities? We hypothesized that the microbiome from inoculum associated with the organic farming soil under historical climate stress contains higher diversity and beneficial microbes that can positively impact the growth characteristics of wheat seedlings. These goals were reached by analyzing important growth traits of wheat seedlings, which are crucial for crop establishment (germination rate and time as well as seedlings above-ground biomass) and bacterial communities from inoculum (soil microbial extracts) and seedlings.

## Materials and methods

### Soil sampling

We took advantage of GCEF, which is a large multi-year field experiment in Saxony-Anhalt, Germany, designed to assess the effects of global changes on various ecosystem processes across a range of land-use types and intensities. Detailed information about GCEF is available in Schädler et al. (2019). This experimental field includes 50 plots (400 m^2^ each) arranged in 10 main-plots (5 plots per main-plots). Five different types of land use are established in each main-plot, including (1) conventional farming; (2) organic farming; (3) intensively used grassland that is frequent mowed; (4) extensively used grassland that is moderately mowed; and (5) extensively used grassland that is utilized for moderate sheep grazing. In conventional crop fields, a crop rotation includes winter rape, winter wheat, and winter barley using mineral fertilizers and pesticides (see Schädler et al., 2019 for details). In conventional fields, N fertilization involves multiple applications of calcium ammonium nitrate (40–60 kg/ha). Potassium (K) is supplemented at a rate of 110 kg/ha using potassium chloride, while phosphate (P) is applied at 30 kg/ha as superphosphate each year. On the organic crop fields, winter rape is replaced by legumes (alternating alfalfa and white clover) for the biological nitrogen (N) fixation. In addition, organic fields receive 120 kg/ha of K in the form of patent kali and 45 kg/ha of P as rock phosphate each year. No pesticides have been applied under organic farming.

**Figure 1:**
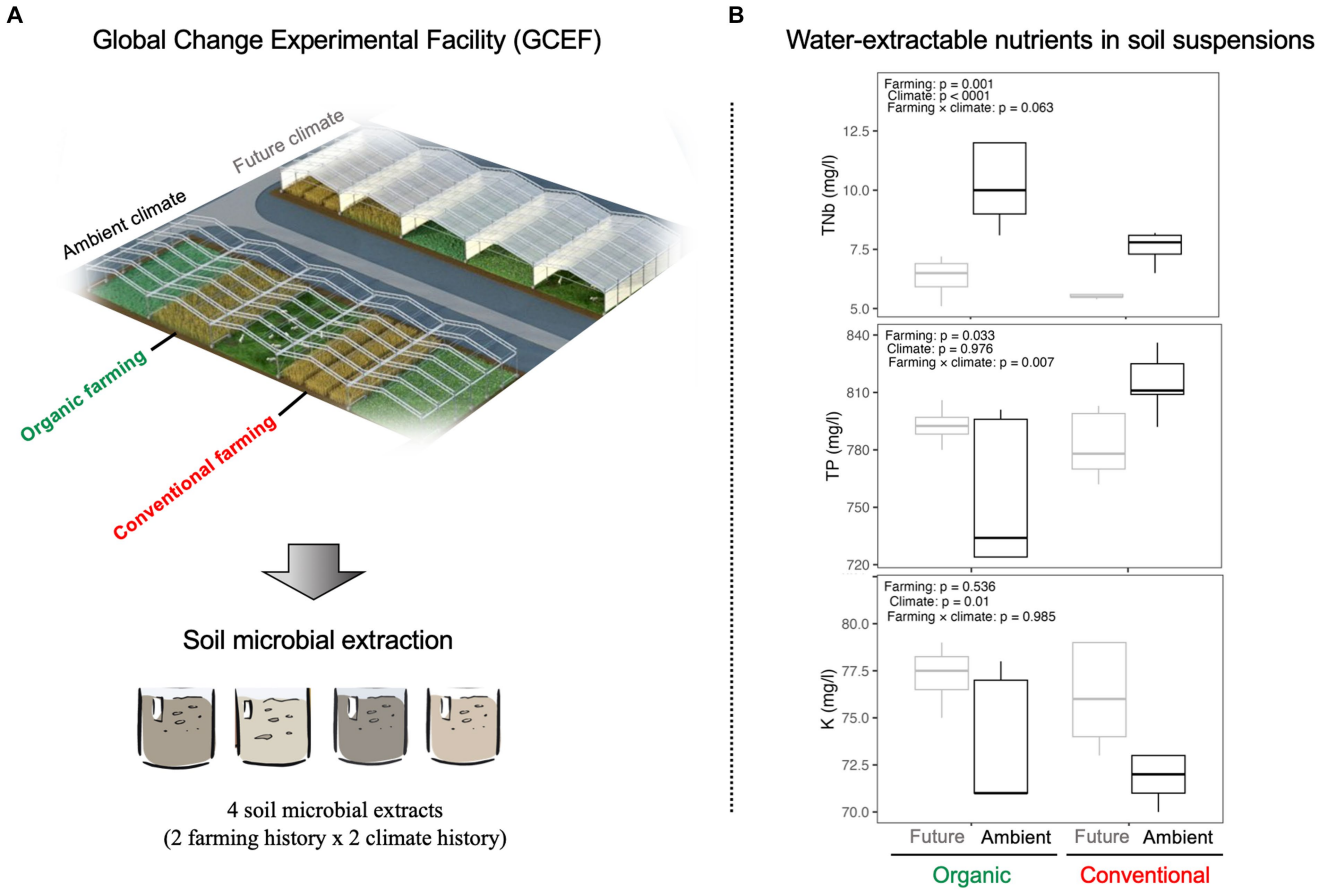
**(A)** GCEF experimental field: 20 soil sub-samples were collected from future and ambient plots associated with organic and conventional farming systems, representing different soil and microbiome stress histories (GCEF Image: Tricklabor Berlin). **(B)** Water-extractable nutrients in soil suspensions. ANOVA was performed to test the impact of different farming (organic vs. conventional) and climate (future vs. ambient) histories on water-extractable nutrients such as total nitrogen (TNb), total phosphorus (TP), and potassium (K).

Half of the main-plots are subjected to the future climate regime projected as a mean scenario for 2070–2100 in such a way that plots are equipped with mobile roofs (5 m height) and side panels together with an irrigation system. In future climate plots, precipitation is reduced by around 20% in the summer months and increased by around 10% during spring and autumn. Furthermore, the shelters and panels are automatically closed from sunset to sunrise on these plots to increase the temperature in all seasons during the night up to 2.5°C with a mean increase throughout the year by ∼0.6°C. The rest of the blocks are exposed to the ambient climate. The climate manipulations in the GCEF started in the spring of 2014. Soil samples were collected in August 2021 from future and ambient plots associated with organic and conventional farming systems, representing different soil and microbiome stress histories (Figure 1A). For both types of crop fields, the previous crop on the plots was winter wheat (RGT Reformed). Twenty soil sub-samples (2 farming history × 2 climate history × 5 replicates) were collected (0.5–0.6 kg from each replicate) from the topsoil layer (0–15 cm depth), mixed (to have one representative sample), and then transferred to the lab. This resulted in ~3 kg of fresh soil for each soil history type. The selected soils have similar soil types and textures but differ in terms of farming and climate histories. All soil samples were kept moist and cold (at 4°C) till the beginning of the experiment.

### Extraction of the soil microbiome

From each soil history type, microbial extractions were carried out by mixing 5 g of soil (taken randomly from 3 kg of fresh soil) with 45 mL of sterilized PBS buffer in a 250 mL autoclaved Erlenmeyer flask for one hour on the shaker at 150 rpm speed. Next, soil suspensions were transferred into 50 mL Falcon tubes and placed into the centrifuge for 5 min at 2,000 rpm to separate microbial suspension from soil particles. This procedure was repeated four times per soil history type and then microbial extractions pooled together before inoculating the seeds. 1 ml from each of these four microbial extractions was immediately taken and stored at −20°C for downstream analysis (e.g., DNA extraction and sequencing assays). We previously demonstrated that such microbial extraction method led to similar microbial communities in the soil before extraction and microbial suspensions (Giard-Laliberté et al., 2019), which was the case in other studies (Walsh et al., 2021). To check for water-extractable soil nutrients, soil suspensions were sent to Eurofins Umwelt Nord GmbH (Göttingen, Germany) to measure total nitrogen (TNb), total phosphorus (TP), and K as the main nutrients used in GCEF. In addition, to have an idea about what proportion of bacteria can survive and grow on agar media (LB and R2A), we prepared a serial dilution from each soil extract, which allowed us to detect and extract distinguishable bacterial colonies (based on the size, color, and shape of each colony). We then cultured and identified these colonies based on Sanger sequencing of the 16S (LGC Genomics GmbH, Berlin, Germany). These results are shown in Supplementary Figure S1.

### Experimental design

Seeds of wheat (*Triticum aestivum* L.) were used in this study. In order to isolate the influence of soil microbial extract from the effects of edaphic and climatic factors, we grew seeds on sterilized microboxes containers (Paungfoo-Lonhienne et al., 2010; Helletsgruber et al., 2017) in a growth chamber. To inoculate the wheat seeds with the soil microbial extracts, microboxes were sterilized and filled with 200 mL of Murashige Skoog plant agar (1.1 g Murashige Skoog from Duchefa, 5.5 g plant agar per liter distilled water). We did not surface sterilize the seeds before microbial inoculation as we wanted to take into account the possible interactions between the indigenous seed and inoculum microbiomes that would eventually shape seedlings’ bacterial communities. Six seeds were placed in a sterile microbox where each seed was treated with 400 μL of soil microbial extracts by pipetting liquid on the seed and its surrounding area. Control (non-inoculated) seeds received 400 μL of sterile PBS buffer. Therefore, this experiment consists of 5 treatments (four soil microbial extracts plus PBS buffer as control). The 400 μL volume was selected based on a pre-test, where we used different amounts of water required for the seed to germinate in sterilized boxes without producing a water layer on top of agar media. The sterile microboxes were placed in the growth chamber under controlled conditions of 16:8 h (light: dark cycle), 22–24°C, and 800 μmol m^−2^ s^−1^ photon flux density. Each treatment was replicated 10 times in a randomized design (5 treatments × 10 replicates = 50 pots). Germination rates and germination times were measured over a period of 6 days. The germination rate was expressed as a percentage where we counted the number of germinated seeds in each treatment and divided it by the total number of planted seeds (6), multiplied by 100. At the end of the experiment (8th day), the plants were carefully removed from the container, the plant agar was discarded, and the seed and roots were cut with a sterile scalpel. The fresh weight of the above-ground biomass (shoot samples) of each seedling was measured for individual plants in each microbox (Supplementary data S1). Following biomass measurements, shoot samples were stored at −20°C prior to DNA extraction. Given the fact that the initial 8 days post-germination can be crucial for wheat seedlings as they undergo significant physiological changes that could provide early indicators of successful establishment in the later development stage, this time was chosen to measure seedling above-ground biomass.

### DNA extraction, amplicon sequencing, and data processing

For extracting DNA from seedlings, 5 replicates (out of 10) were randomly selected within each treatment, where shoot samples of all seedlings in each microbox were ground into a powder using liquid nitrogen with a mortar and pestle. DNA was extracted from 0.1 g of seedlings or 1 mL solution of microbial extract using a phenol-chloroform extraction method (Dellaporta et al., 1983). This resulted in 25 DNA samples from seedlings and 16 samples from soil microbial extracts. Further information on DNA extraction is presented in Azarbad et al. (2018). DNA samples were sent to LGC Genomics GmbH (Berlin, Germany) for libraries preparation and Illumina MiSeq (paired-end) sequencing. For the bacterial 16S rRNA gene, the V3–V4 region was amplified using primers 341F (CCTACGGGNGGCWGCAG) and 785R (GACTACHVGGGTATCTAAKCC). Sequence-specific peptide nucleic acid (PNA) clamps, as recommended by Fitzpatrick et al. (2018), were used in order to block the amplification of plant-derived DNA and reduce host mitochondrial and chloroplast DNA during amplification. Sequence data were analysed following procedures described by Walsh et al. (2021). Raw reads were processed using a DADA2-based bioinformatic pipeline version 1.10.1 (Callahan et al., 2016) in the R software. Briefly, primer sequences were removed, and reads were truncated to 250 and 200 bp for forward and reverse reads (maxEE = c(2,2), maxN = 0, truncQ = 2, rm.phix = TRUE), respectively. Chimeric sequences were identified and removed with the removeBimeraDenovo function of DADA2. For taxonomic affiliations of the resulting amplicon sequence variants (ASV), a naive Bayesian classifier was performed based on the SILVA database v138. ASVs unassigned at the phylum level, together with chloroplast and mitochondrial reads, were removed from the dataset. Before further analysis, the following filtering criteria were applied: samples should have more than 1,000 ASV reads, and any ASVs with less than five reads in a given sample were removed. Furthermore, any ASV that was found in only one sample was discarded from the data set. For the calculation of alpha diversity, the data were normalized on the basis of sequencing depth. For beta diversity, we normalized the data set based on the relative abundance of ASVs in each sample (Walsh et al., 2021).

### Statistical analyses

Statistical analyses were performed using R software (v 4.4.2, The R Foundation for Statistical Computing). An analysis of variance (ANOVA) was conducted to determine the potential impact of farming and climate histories on nutrients in soil extracts, germination rate and times, seedling biomass, and bacteria Shannon’s diversity index, considering both direct and interactive effects. We conducted principal coordinate analyses (PCoA) and the Permanova test (through the ‘*adonis*’ function) to evaluate the effect of experimental factors on the bacterial community composition of soil microbial extract and wheat seedlings. Our analysis was based on the relative abundance of ASVs, using Bray–Curtis dissimilarity. For each farming and climate history, to determine if soil microbial extracts are more similar to inoculated seedlings compared to non-inoculated control plants, we calculated the Bray–Curtis dissimilarity for each extract and corresponding plants and tested the significance of the differences using the Wilcoxon ranked test. Additionally, we compared the number of shared and unique ASVs between the bacterial communities of inoculated seedlings and their respective inoculum (soil microbial extracts) to the number of shared and unique ASVs between the communities of non-inoculated control plants and the same inoculum (Giard-Laliberté et al., 2019). We also used ANOVA to analyze the impact of each experimental factor on the relative abundance of dominant bacteria at the corresponding taxonomic level (mean relative abundance of >1%). After excluding unclassified families, a heatmap (based on the Euclidean distance as a dissimilarity measure) was created to display the variation in the relative abundance of the dominant bacterial families whose relative abundance showed significant changes due to experimental factors (based on ANOVA results). Finally, Spearman’s rank correlation (*p* < 0.05, Benjamini-Hochberg corrected) was used to analyze the correlation between the growth characteristics of wheat seedlings and nutrients and bacterial families in soil microbial extracts.

## Results

### Water-extractable nutrients in soil suspensions

Soil extracts from ambient climates contained a higher amount of TNb as compared to future climates under both farming management (Figure 1B). Soil suspension from conventional farming under ambient climate had the highest concentration of TP (significant farming × climate interaction; Figure 1B). Moreover, K showed a higher concentration in future climate, independent of farming systems (Figure 1B).

### Seedlings growth parameters

**Figure 2:**
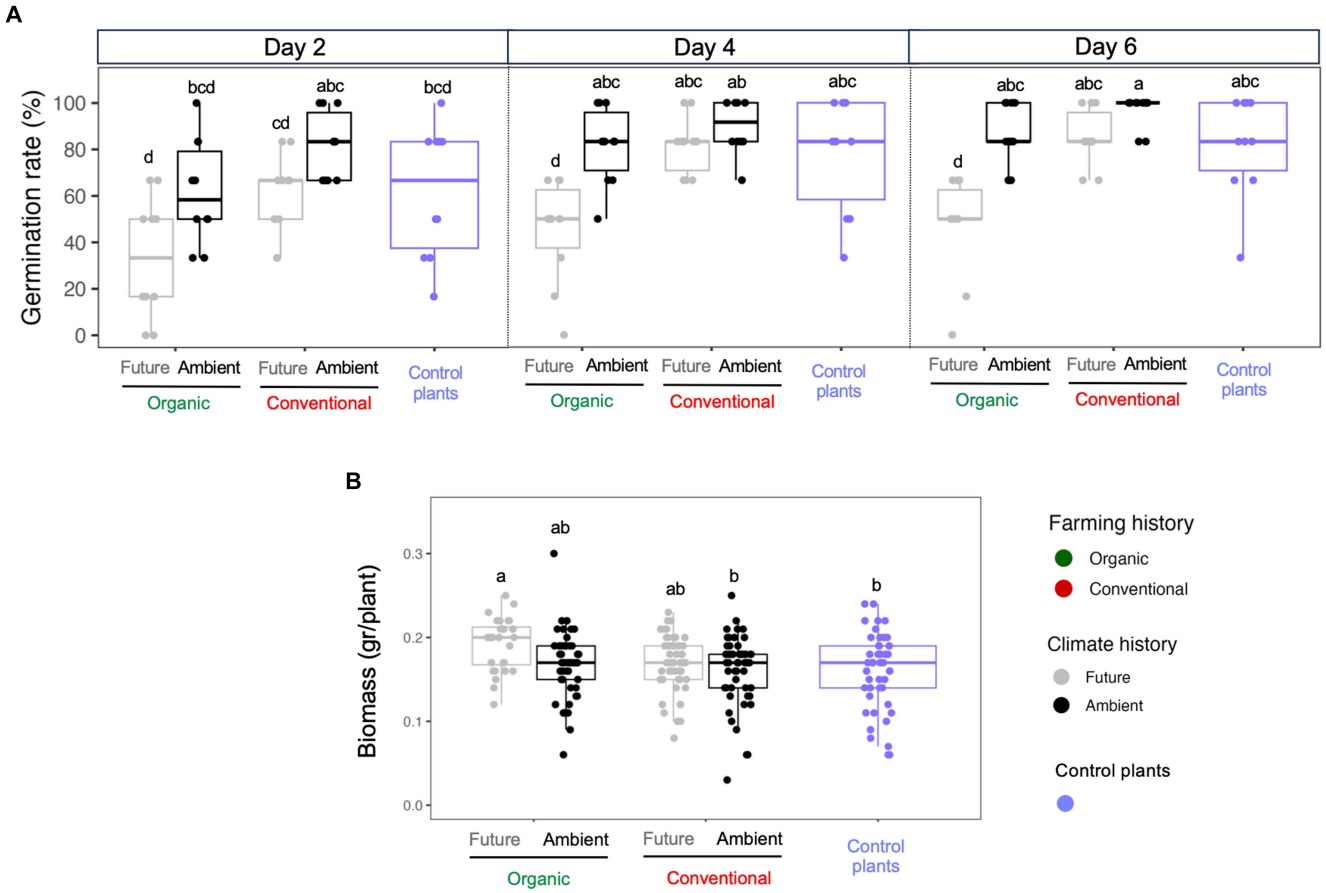
**(A)** Germination rates and germination times and **(B)** the above-ground biomass of each seedling where seeds were inoculated with soil microbial extracts from different farming (organic vs. conventional) and climate (future vs. ambient) histories and non-inoculated control plants. Letters represent significant differences based on *post hoc* Tukey’s test.

The climate history of microbial extracts was the main source of variation explaining wheat seed germination (*F* = 63.16; Supplementary Table S1), with seeds inoculated with microbes exposed to ambient climate showed, in general, the highest germination rate (day 6 in Figure 2A). In addition, seeds inoculated with soil microorganisms extracted from conventional farming, with a history of ambient climate, exhibited the highest and fastest rate of germination when compared with other treatments (Figure 2A). Conversely, microorganisms derived from organic farming and exposed to future climate recorded the lowest and slower rate of germination in all treatment combinations (Figure 2A; Supplementary Table S1). As for seedlings’ biomass, we observed that seeds inoculated with soil microbial extract from organic farming under future climate produced the highest biomass (on average 0.192 gr/plant; Figure 2B). On the other hand, non-inoculated seeds and those inoculated with soil extract from conventional farming under ambient climate produced the lowest biomass (on average 0.162 and 0.160 gr/plant, respectively; Figure 2B).

### The diversity and structure of bacterial communities in soil extracts and seedlings

**Figure 3:**
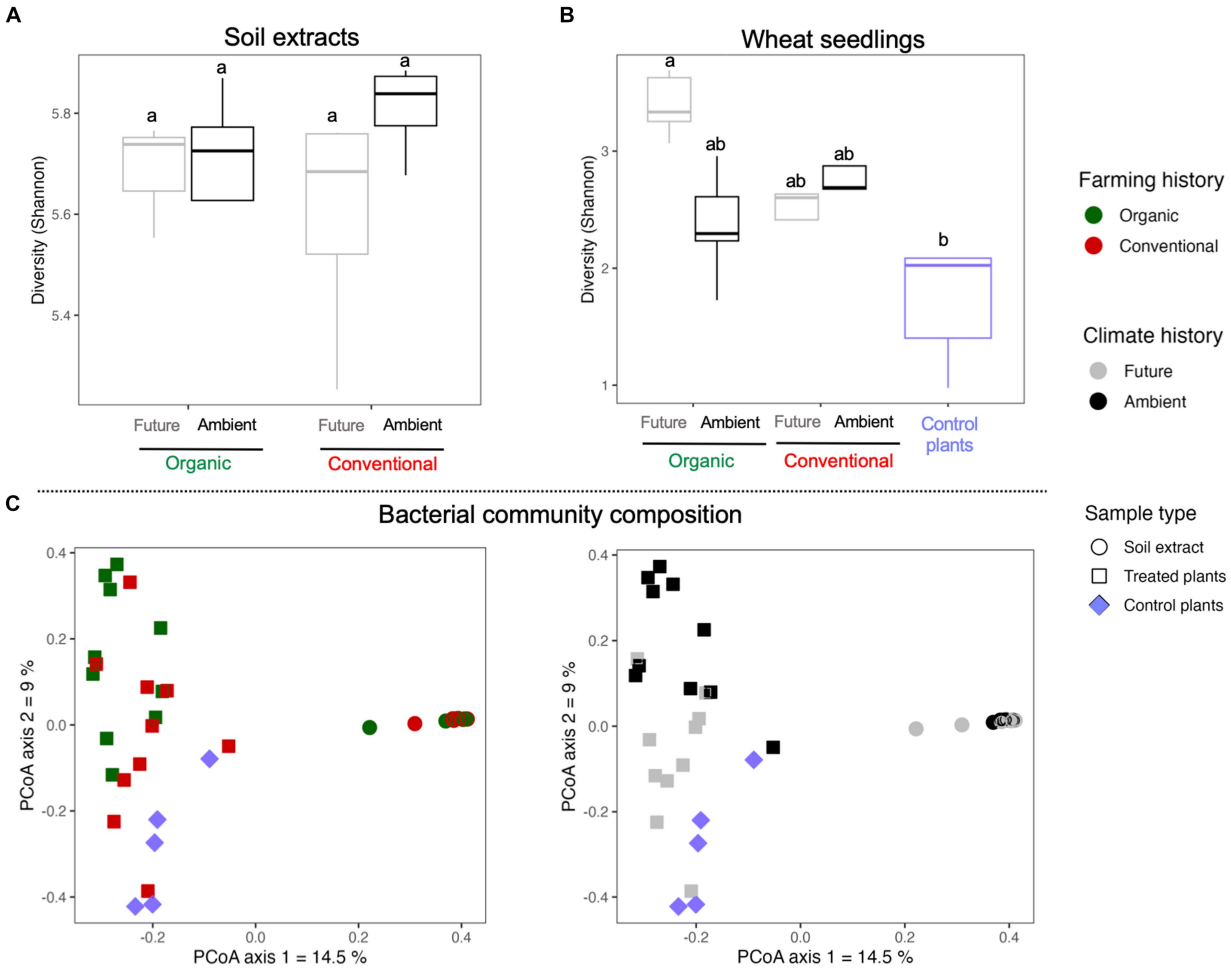
Bacterial Shannon diversity of **(A)** soil microbial extracts and **(B)** wheat seedlings. **(C)** Principal coordinate analyses (PCoA) of Bray–Curtis dissimilarity visualizing the impact of sample type, farming, and climate histories on bacterial community composition associated with soil extracts and wheat seedlings.

No significant independent or interactive effects of farming and climate histories were observed for the bacterial diversity in the soil extracts (Figure 3A and Table 1). However, seeds inoculated with soil microbial extracts from organic farming showed higher bacterial diversity associated with seedlings than other treatments (Figure 3B and Table 1). Furthermore, the impact of soil microbe’s climate history was only evident in organic farming, where seedlings that were exposed to soil microbes with a history of future climates displayed a higher level of bacterial diversity (significant interactive effect of farming and climate; Figure 3B and Table 1).

**Table 1:** ANOVA test for the effects of farming and climate histories and their interactions on bacterial Shannon diversity and bacterial communities of soil extracts and seedlings.

	Shannon diversity				Community structure			
	Soil extracts		Seedling		Soil extracts		Seedling	
	*F*-value	*p*-value	*F*-value	*p*-value	*F*-value	*p*-value	*F*-value	*p*-value
Farming	0.062	0.808	5.305	**0.014**	1.826	0.087	3.421	**0.001**
Climate	1.381	0.265	2.206	0.153	4.672	**0.001**	3.986	**0.001**
Farming × Climate	1.468	0.251	7.764	**0.011**	1.946	0.058	2.505	**0.001**

Farming refers to the history of soil microbes extracted from organic and conventional agriculture, and climate refers to the history of soil microbes extracted from future and ambient climate under each farming.

We conducted a Principal Coordinate Analysis (PCoA) to see how the bacterial community structure was influenced by the experimental factors, considering both the soil microbial extracts and wheat seedlings. These results revealed noticeable differences in the bacterial communities between sample types where soil extract and seedlings clustered separately along the first axis of the PCoA plots (Figure 3C). The bacterial communities of the seedlings were separated by experimental factors along the second axis of the PCoA plots (Figure 3C). Due to strong effect of sample types on bacterial communities, we conducted Permanova analyses for soil extracts and seedlings samples separately to confirm PCoA patterns (Table 1). The results of the Permanova analyses indicated that climate history had a significant direct effect on the bacterial community structure of soil microbial extracts (Table 1). As for the seedlings, farming and climate histories of soil microbes had significant direct and interactive effects on the restructuring of the seedling bacterial community. Climate history was found to be a major source of variation (higher *F* value in Table 1) with a clear gradient from ambient to future to non-inculcated control plants along the second axis of the PCoA plots, which was more pronounced under organic farming (Figure 3C).

### Similarity and shared ASVs between soil extracts and seedlings

We further tested whether there was a greater similarity between soil microbial extracts and inoculated seedlings compared to non-inoculated control plants. The Bray–Curtis dissimilarity was calculated for each soil extract and its corresponding inoculated plants, as well as for the soil extract and control plants. Results showed that for organic farming, seedlings that were inoculated with microbes sourced from the soil with past experience of future climate were more similar to each other than control plants (nonsignificant *p*-value for PC2; Supplementary Table S2). There were also more shared ASVs between soil extract and seedlings from future climate (16 ASVs; Supplementary Figure S2A) than ambient climate. However, for plants introduced to microbes from conventional farming, seedlings showed more similarity (Supplementary Table S2) and shared ASVs (23 ASVs; Supplementary Figure S2B) with their respective soil extract only for the ambient climate.

### The composition of bacterial communities

We examined changes in the composition of bacterial communities at the phyla/class (top 10; Supplementary Figure S3) and order levels (top 25; Supplementary Figures S4,S5) for soil extracts and inoculated and non-inoculated plant samples. At the order levels, in the case of soil extracts, the relative abundance of bacteria associated with *IMCC26256* (belonged to *Acidobacteriota* phyla) and *Xanthomonadales* under conventional farming was significantly higher than for organic farming (direct effect of farming; Table 2 and Supplementary Figure S4). The relative abundance of *Haliangiales* under ambient climate was significantly higher than for future climate, regardless of farming history (direct effect of climate; Table 2 and Supplementary Figure S4). When it comes to treated plants, the relative abundances of *Cytophagales and Sphingobacteriales* were higher in the future than ambient climate only for organic farming (Table 2 and Supplementary Figure S5). The relative abundances of *Burkholderiales and Xanthomonadales* were higher in the future climate, irrespective of the farming (Table 2 and Supplementary Figure S5).

**Table 2:** ANOVA test for the significant effects of farming and climate histories and their interactions on bacteria at order and family levels for soil extracts and seedlings (only significant values are shown).

Order level	Farming		Climate		Farming * Climate	
	*F*-value	*p*-value	*F*-value	*p*-value	*F*-value	*p*-value
Soil extracts						
*Haliangiales*	2.586	0.136	11.40	**0.006**	0.326	0.579
*IMCC26256*	14.17	**0.003**	3.358	0.094	0.914	0.360
*Vicinamibacterales*	2.293	0.158	0.030	0.866	5.807	**0.035**
*Xanthomonadales*	11.09	**0.007**	3.519	0.087	0.687	0.425
Treated plants						
*Bacillales*	1.141	0.301	2.069	0.170	6.029	**0.026**
*Burkholderiales*	0.000	1.000	5.626	**0.031**	1.329	0.266
*Cytophagales*	8.578	**0.010**	7.991	**0.012**	7.991	**0.012**
*Sphingobacteriales*	3.434	0.082	19.56	**0.000**	5.265	**0.036**
*Xanthomonadales*	1.166	0.296	6.058	**0.026**	0.000	0.983
Family level						
Soil extracts						
*Anaerolineaceae*	16.10	**0.002**	5.436	**0.040**	0.140	0.716
*BIrii41*	10.90	**0.007**	2.707	0.128	0.845	0.378
*Bdellovibrionaceae*	2.900	0.117	5.091	**0.045**	0.338	0.573
*Caulobacteraceae*	1.694	0.220	5.101	**0.045**	0.007	0.935
*Chthoniobacteraceae*	8.600	**0.014**	2.822	0.121	0.257	0.622
*Comamonadaceae*	3.349	0.094	5.187	**0.044**	0.364	0.559
*Haliangiaceae*	2.586	0.136	11.403	**0.006**	0.326	0.579
*Longimicrobiaceae*	8.385	**0.015**	14.216	**0.003**	25.55	**0.000**
*Micrococcaceae*	1.086	0.320	4.934	**0.048**	0.761	0.402
*Micropepsaceae*	10.46	**0.008**	14.238	**0.003**	4.771	**0.051**
*Nannocystaceae*	6.277	**0.029**	0.391	0.544	0.060	0.812
*Opitutaceae*	2.005	0.184	12.75	**0.004**	0.927	0.356
*Rhizobiaceae*	0.022	0.885	2.741	0.126	5.615	**0.037**
*Rhizobiales Incertae Sedis*	3.345	0.095	0.151	0.705	5.978	**0.033**
*Rhodanobacteraceae*	3.872	0.075	6.781	**0.025**	0.031	0.862
*Sutterellaceae*	8.346	**0.015**	9.248	**0.011**	11.73	**0.006**
*Verrucomicrobiaceae*	11.48	**0.006**	0.002	0.968	6.05	**0.032**
*Xanthomonadaceae*	10.88	**0.007**	1.497	0.247	1.217	0.294
Treated plants						
*Alcaligenaceae*	0.106	0.749	5.468	**0.033**	0.113	0.741
*Beijerinckiaceae*	7.185	**0.016**	9.626	**0.007**	4.745	**0.045**
*Burkholderiaceae*	8.308	**0.011**	4.352	**0.053**	4.257	0.055
*Devosiaceae*	8.323	**0.011**	1.442	0.247	1.442	0.247
*Enterobacteriaceae*	1.772	0.202	28.746	**0.000**	0.218	0.647
*Erwiniaceae*	1.045	0.322	4.557	**0.049**	1.709	0.210
*Micrococcaceae*	1.853	0.192	2.333	0.146	4.293	**0.055**
*Nocardiaceae*	6.156	**0.025**	2.438	0.138	2.258	0.152
*Pseudomonadaceae*	0.418	0.527	6.304	**0.023**	0.543	0.472
*Sphingobacteriaceae*	3.434	0.082	19.564	**0.000**	5.265	**0.036**
*Spirosomaceae*	8.578	**0.010**	7.991	**0.012**	7.991	**0.012**
*Xanthobacteraceae*	4.849	**0.043**	3.394	0.084	3.394	0.084
*Xanthomonadaceae*	1.698	0.211	7.753	**0.013**	0.298	0.593
*Yersiniaceae*	0.676	0.423	0.676	0.423	4.644	**0.047**

**Figure 4:**
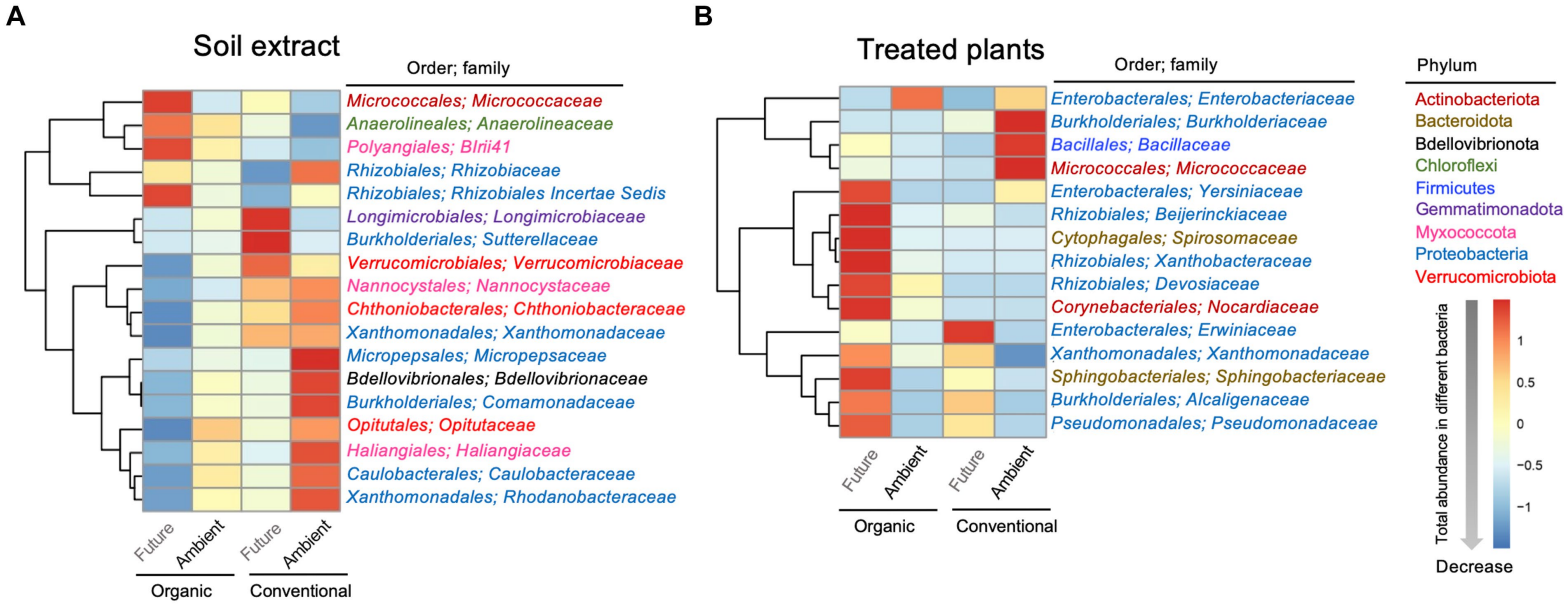
Heatmaps showing the abundance distributions of dominant bacterial families [in soil extract **(A)** and in the treated plant **(B)**] whose relative abundance was significantly affected by experimental factors. The bacterial phylum and order that each family belonged to are shown. Heatmaps are color-coded based on row z-scores with default clustering methods (Euclidean distances).

To look at finer taxonomical levels, a heatmap was used to visualize the variations in the relative abundances of dominant members of the bacterial communities at family levels (top 70; Table 2 and Figure 4) that showed significant differences in terms of experimental factors. As for soil microbial extracts, the relative abundances of the dominant bacterial families varied significantly across farming and climate histories (Figure 4A and Table 2). The effect of farming was significant for *BIrii41* (belonged to *Myxococcota* phyla), *Chthoniobacteraceae*, *Nannocystaceae*, *Xanthomonadaceae* (Figure 4A and Table 2). The effect of climate was significant for *Bdellovibrionaceae*, *Caulobacteraceae*, *Comamonadaceae*, *Haliangiaceae*, *Micrococcaceae*, *Opitutaceae*, and *Rhodanobacteraceae* (Figure 4A and Table 2). The interaction effect between farming and climate was significant for several families such as *Longimicrobiaceae*, *Micropepsaceae*, *Rhizobiaceae*, *Rhizobiales Incertae Sedis*, *Sutterellaceae*, *Verrucomicrobiaceae* (Figure 4A and Table 2). For example, the relative abundance of *Rhizobiales Incertae Sedis* enriched under future climate, which was evident only for organic farming. An opposite pattern was observed for *Micropepsaceae* and *Rhizobiaceae* where soil extracts from conventional farming under ambient climate showed higher abundance than other treatments.

As for treated plants, the abundances of *Bacillaceae* and *Micrococcaceae* were significantly higher under ambient climate only for conventional farming. On the other hand, the abundances of *Yersiniaceae, Beijerinckiaceae* and *Spirosomaceae* were significantly higher under future climate only for organic farming (significant farming × climate interaction; Figure 4B; Table 2). The relative abundance of *Sphingobacteriales* was higher under future climates in both farming histories, with a more pronounced pattern for organic farming (Figure 4B and Table 2).

### Correlation between seedlings growth parameters with bacterial and nutrients in soil extracts

We performed Spearman’s rank correlation analyses to explore possible links between seedlings’ growth parameters with bacterial families and nutrients in soil extracts (Supplementary Table S4). For bacteria, correlation was performed only for those families whose abundances were significantly affected by experimental factors. Since the most interesting differences in germination rate and biomass were observed between organic farming under the future and conventional farming under ambient climates, here the correlation results are centered on these two treatments. In the context of conventional farming, we observed a positive correlation between the germination rate and the abundance of *Micropepsaceae* (Spearman’s rho = 0.84) and *Rhizobiaceae* (Spearman’s rho = 0.462). By looking at the relative abundance data (Figure 4A) and germinate rate (Figure 2A), we can see that such a positive correlation can be explained by climate history in such a way that higher relative abundance of these two families in soil extract from ambient climate positively correlated with higher germination rates in this treatment. For organic farming, the higher relative abundance of *Rhizobiales Incertae Sedis* in soil extract from future climate correlated positively with the higher seedling biomass (Spearman’s rho = 0.459). In both farming histories, higher TNb concentrations in ambient climate showed a positive correlation with seed germination rates (organic: Spearman’s rho = 0.720; conventional: Spearman’s rho = 0.459). This trend was observed for TP but only in conventional farming (Spearman’s rho = 0.384). Higher K levels in organic farming under future climate correlated positively with seedling biomass (Spearman’s rho = 0.161).

## Discussion

In this study, we took advantage of a large multi-year field experiment where we collected soil from organic and conventional fields with different climate conditions to extract microbes to inoculate wheat seeds. The main aim was to study whether water-extractable soil microbiomes from different agricultural practices and climate conditions impact the germination rate and time as well as wheat seedlings aboveground biomass and their associated bacterial communities. Based on agar medium assays, we showed that seeds inoculated with microbial extracts from conventional farming under ambient climate exhibited the highest and fastest germination rate. In contrast, seeds exposed to microbes from organic farming under future climate revealed the lowest and slower germination rate but resulted in higher aboveground biomass and bacterial diversity associated with seedlings. These results suggest that under future climate scenarios, organic agriculture may promote more resilient plant-microbe interactions, as evidenced by the higher bacterial diversity and above-ground biomass in wheat seedlings.

Soil collected from experimental field plots under future climate experienced more changes in precipitation and increased temperature (on average by 0.55°C daily mean temperature and 1.1°C minimum temperatures) in comparison with ambient plots (Schädler et al., 2019). Such an increase in temperature led to an extended frost-free period in stress-treated plots (Schädler et al., 2019). Therefore, a combination of these stress factors, which was repeated over a period of 10 years, can influence soil-associated microbial community and structure, as well as abiotic soil characteristics, and these effects can be amplified or mitigated depending on the type of farming management. In our study, certain important bacterial orders and families in soil microbial extracts showed distinct abundance patterns in relation to farming and climate histories. Specifically, soil extracted from conventional farming under ambient climate showed an increase in the abundance of *Micropepsaceae* and *Rhizobiaceae*. Members of *Rhizobiaceae* have been reported to produce gibberellins (known to enhance seed germination) as an important phytohormone (Gopalakrishnan et al., 2015), which could potentially promote seed germination. On the other hand, soil extracts from organic farms under future climate conditions contained a higher abundance of the *Rhizobiales Incertae Sedis* group, which is recognized for its nitrogen-fixing capabilities (Garrido-Oter et al., 2018), likely to contribute to increasing seedling biomass. Our results agree with an earlier report by Quattrone et al. (2024) that the higher abundance of *Rhizobiales Incertae Sedis* families in the rhizosphere soils positively affected the biomass of maize seedlings (Quattrone et al., 2024).

Furthermore, we showed that the soil microbial extracts used as an inoculation source significantly altered the bacterial communities associated with seedlings. This finding is similar to previous research, indicating that soil microorganisms play an important role in the colonization of plant-associated microbiomes (Hardoim et al., 2012; Grady et al., 2019; Azarbad et al., 2020; Walsh et al., 2021; Azarbad, 2022). For example, Walsh et al. (2021) showed that soil microbial extracts (where soils were collected from 219 different soil types across the United States) had a significant impact on shaping the microbiomes of wheat seedlings following a week of growth after seeds were inoculated. Here, we showed that the relative abundance of several bacterial families (e.g., *Yersiniaceae, Beijerinckiaceae Spirosomaceae*, and *Sphingobacteriales*) was notably higher in seedlings that were inoculated with soil microbial extracts from organic farming under future climate. As part of designing synthetic microbial communities to improve crop yield, Gonçalves et al. (2023) demonstrated that the *Beijerinckiaceae* species encompass a comprehensive set of genes responsible for phosphate transport, homeostasis, and degradation, as well as the production of siderophores and the synthesis of indole-3-acetic acid (IAA). Such beneficial bacteria from inoculum can potentially improve the health of indigenous seed microbiomes (and subsequently seedlings) by replacing non or less-beneficial bacterial species that are already present inside and outside of the seeds or act synergistically with other beneficial bacteria to colonize seedlings. If that is the case, such interactions between indigenous and inoculum-introduced bacteria could enhance the overall functioning of the community, leading to beneficial effects for the host plants (Afkhami, 2023; Allsup et al., 2023). Indeed, seeds inoculated with microbes that have been exposed to future climate scenarios within organic farming displayed a higher bacterial diversity associated with seedlings, positively linked with an increase in seedling biomass. Our results agree with previous research based on the GCEF field that the organic agricultural practice under future climate significantly enhanced total AMF richness colonizing wheat roots (Wahdan et al., 2021). Such findings support the idea that organic agricultural practices not only mitigate the adverse effects of climate change but also promote the diversity of seedling-associated bacteria.

It is important to highlight that the combined effect of climate and agricultural management may not only affect soil biotic factors but also abiotic properties. Therefore, besides microbial effects, water-extractable nutrients from the soil, even though diluted, may also influence seedlings’ growth parameters. We observed that a higher germination rate of seed inoculated with microbes from conventional farming under ambient climate correlated positively with total nitrogen and phosphorus levels, but this was not reflected in a growth advantage for seedlings. Some studies have shown that dry environments (such as those under future climates) lead to higher levels of K in the soil than moist environments, which is due to the fact that K is more quickly leached from litter than other nutrients (e.g., N and P) and thus has a much shorter residence time in soil organic matter (Sardans and Peñuelas, 2021). This can explain the higher K level that we observed under the future climate, which showed a positive correlation with higher seedling biomass in organic farming. These results reveal a complex interaction between farming management and climate scenarios with contrasting effects on soil biotic and abiotic factors. Therefore, in this study, it is not possible to conclude if the observed patterns in seedlings’ growth parameters are due to microbes or nutrient levels in soil extracts. However, since higher N and P levels were not projected in higher seedlings growth put more weight towards the idea that the beneficial impact of soil microbes on plant growth may be more critical, at least partially, than certain nutrients. Having the list of culturable bacterial species from soil extracts (shown in Supplementary Figure S1), the next step is to build synthetic bacterial communities to tease apart the effect of microbes from those abiotic factors on seedlings’ growth parameters. This will help to confirm our results, which are based on the “whole community” inoculation approach.

It is important to note that there are several limitations that should be taken into account when interpreting the results of our study. The conditions defined under organic and conventional farming in GCEF are based on European regulations (particularly in Germany), thus limited to specific regions, and are not necessarily extrapolated to other environments or countries. The type of assay designed to evaluate the effect of microbes on inoculated wheat plants is not representative of real field conditions. Only a fraction of soil bacteria (and not fungi) would be able to grow and survive in sterile agar boxes, and thus, only culturable bacteria may affect the seedling’s growth parameters. Future studies under soil mesocosms or field conditions will help to conform our findings by taking into account more wheat genotypes and monitoring different morphological and physiological plant traits (including seed quantity and quality at harvest), together with microbial parameters (including both bacteria and fungi) throughout the growing season.

## Conclusion

Our results highlight the importance of organic farming practices in promoting bacterial diversity and potentially enhancing seedlings’ biomass. The next step is to answer open questions regarding the details behind the functioning of the soil and plant microbiome, which are related to benefits to the plant under different histories of agricultural practices and climate. This knowledge can support agricultural strategies for sustainable and resilient food production systems, especially in the face of climate uncertainty.

## Data availability statement

Fastq files are deposited in the NCBI Sequence Read Archive (the BioProject accession PRJNA1085030).

## Author contributions

MO: Data curation, Methodology, Writing – review & editing. RS: Writing – review & editing, Data curation, Methodology. GA: Data curation, Methodology, Writing – review & editing. MS: Writing – review & editing, Resources. HA: Writing – review & editing, Funding acquisition, Supervision, Writing – original draft.
