# Supplementary material for: The stress history of soil bacteria under organic farming enhances the growth of wheat seedlings
Source: Front Microbiol. 2024 Mar 21;15:1355158. doi: 10.3389/fmicb.2024.1355158 (PMC10993729; doi:10.3389/fmicb.2024.1355158)
Supplement: Supplementary file 1 [file Table_1.DOCX]

**Table S1.** ANOVA test for the effects of farming history, climate history, and their interactions on seed germination and seedlings biomass.

|  | Germination | | Biomass | |
| --- | --- | --- | --- | --- |
| Factors | F-value | p-value | F-value | p-value |
| Farming | 30.72 | **<0.001** | 3.165 | **0.044** |
| Climate | 63.16 | **<0.001** | 5.309 | **0.022** |
| Day | 9.95 | **<0.001** | - | - |
| Farming × Climate | 15.05 | **<0.001** | 2.534 | 0.112 |
| Farming × Day | 0.035 | 0.999 | - | - |
| Climate × Day | 0.109 | 0.954 | - | - |
| Farming × Climate × Day | 0.858 | 0.464 | - | - |
|  |  |  |  |  |

**Table S2.** The Bray-Curtis dissimilarity for each microbial extract and corresponding inoculated plants. The significance of the differences was tested using the Wilcoxon ranked test.

|  |  | Treated plants | | Control plants | |
| --- | --- | --- | --- | --- | --- |
| Farming history | Climate history | PC1 | PC2 | PC1 | PC2 |
| Organic | Ambient | 0.016 | 0.016 | 0.016 | 0.016 |
|  | Future | 0.036 | **0.79** | 0.036 | 0.036 |
| Conventional | Ambient | 0.015 | **0.190** | 0.015 | 0.015 |
|  | Future | 0.015 | 0.015 | 0.015 | 0.015 |

|  | Germination rate | | | | Biomass | | | |
| --- | --- | --- | --- | --- | --- | --- | --- | --- |
|  | Organic | | Conventional | | Organic | | Conventional | |
|  | Rho value | P-  value | Rho value | P-  value | Rho value | P-  value | Rho value | P-  value |
| **Bacterial family in soil extracts** |  |  |  |  |  |  |  |  |
| *Micrococcaceae* | -0.596 | **0.000** | -0.459 | **0.000** | 0.211 | **0.000** | 0.040 | 0.402 |
| *Anaerolineaceae* | -0.358 | **0.003** | -0.402 | **0.000** | 0.126 | **0.046** | 0.035 | 0.464 |
| *BIrii41* | -0.358 | **0.003** | -0.115 | 0.349 | 0.126 | **0.046** | 0.010 | 0.834 |
| *Rhizobiaceae* | -0.238 | 0.053 | 0.462 | **0.000** | 0.084 | 0.177 | -0.041 | 0.400 |
| *Rhizobiales Incertae Sedis* | -0.477 | **0.000** | 0.402 | **0.000** | 0.169 | **0.008** | -0.035 | 0.464 |
| *Longimicrobiaceae* | 0.358 | **0.003** | -0.459 | **0.000** | -0.126 | **0.046** | 0.040 | 0.402 |
| *Sutterellaceae* | 0.119 | 0.326 | -0.459 | **0.000** | -0.042 | 0.480 | 0.040 | 0.402 |
| *Verrucomicrobiaceae* | 0.477 | **0.000** | -0.287 | **0.013** | -0.169 | **0.008** | 0.025 | 0.601 |
| *Nannocystaceae* | 0.119 | 0.326 | 0.057 | 0.649 | -0.042 | 0.480 | -0.005 | 0.917 |
| *Chthoniobacteraceae* | 0.477 | **0.000** | 0.230 | 0.048 | -0.169 | **0.008** | -0.020 | 0.675 |
| *Xanthomonadaceae* | 0.477 | **0.000** | 0.000 | 1.000 | -0.169 | **0.008** | 0.000 | 1.000 |
| *Micropepsaceae* | 0.238 | 0.053 | 0.459 | **0.000** | -0.084 | 0.177 | -0.040 | 0.402 |
| *Bdellovibrionaceae* | 0.477 | **0.000** | 0.287 | **0.013** | -0.169 | **0.008** | -0.025 | 0.601 |
| *Comamonadaceae* | 0.238 | 0.053 | 0.402 | **0.000** | -0.084 | 0.177 | -0.035 | 0.464 |
| *Opitutaceae* | 0.715 | **0.000** | 0.287 | **0.013** | -0.253 | **0.000** | -0.025 | 0.601 |
| *Haliangiaceae* | 0.477 | **0.000** | 0.459 | **0.000** | -0.169 | **0.008** | -0.040 | 0.402 |
| *Caulobacteraceae* | 0.477 | **0.000** | 0.345 | **0.003** | -0.169 | **0.008** | -0.030 | 0.530 |
| *Rhodanobacteraceae* | 0.358 | **0.003** | 0.402 | **0.000** | -0.126 | **0.046** | -0.035 | 0.464 |
|  |  |  |  |  |  |  |  |  |
| **Nutrients in soil extracts** |  |  |  |  |  |  |  |  |
| TNb | 0.720 | **0.000** | 0.459 | **0.000** | -0.260 | **0.000** | 0.040 | 0.348 |
| TP | -0.288 | **0.005** | 0.384 | **0.000** | 0.104 | **0.048** | -0.033 | 0.432 |
| K | -0.441 | **0.000** | -0.331 | **0.000** | 0.161 | **0.002** | 0.029 | 0.498 |

**Table S3.** Significant (P < 0.05) Spearman correlations between seedlings growth parameters and bacterial family and nutrients in soil extracts for each farming history.

**
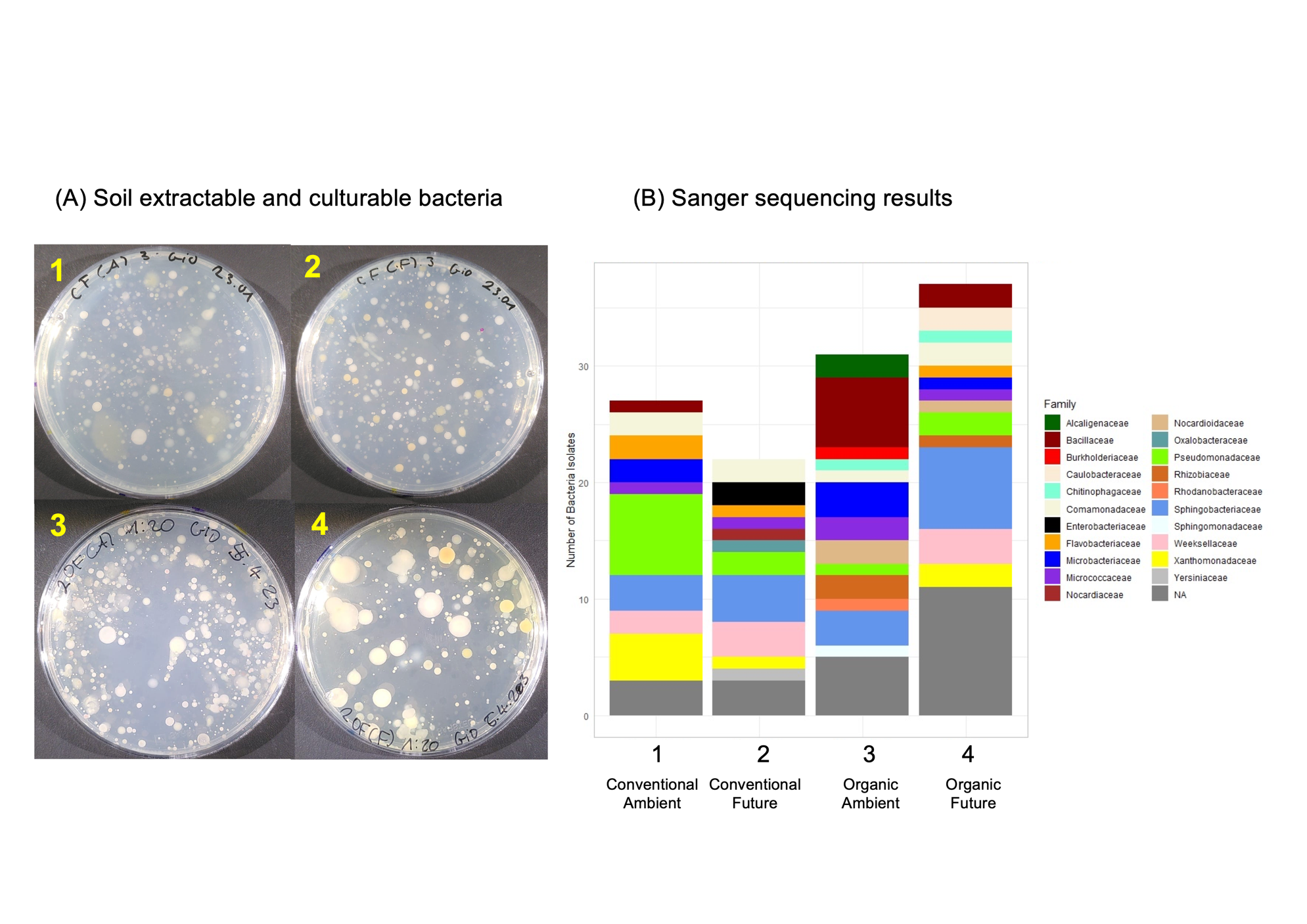
**

**Figure S1.** Different bacterial colonies associated with soil microbial extracts (A). The result of Sanger sequencing of the 16S of detected bacterial colonies (B).


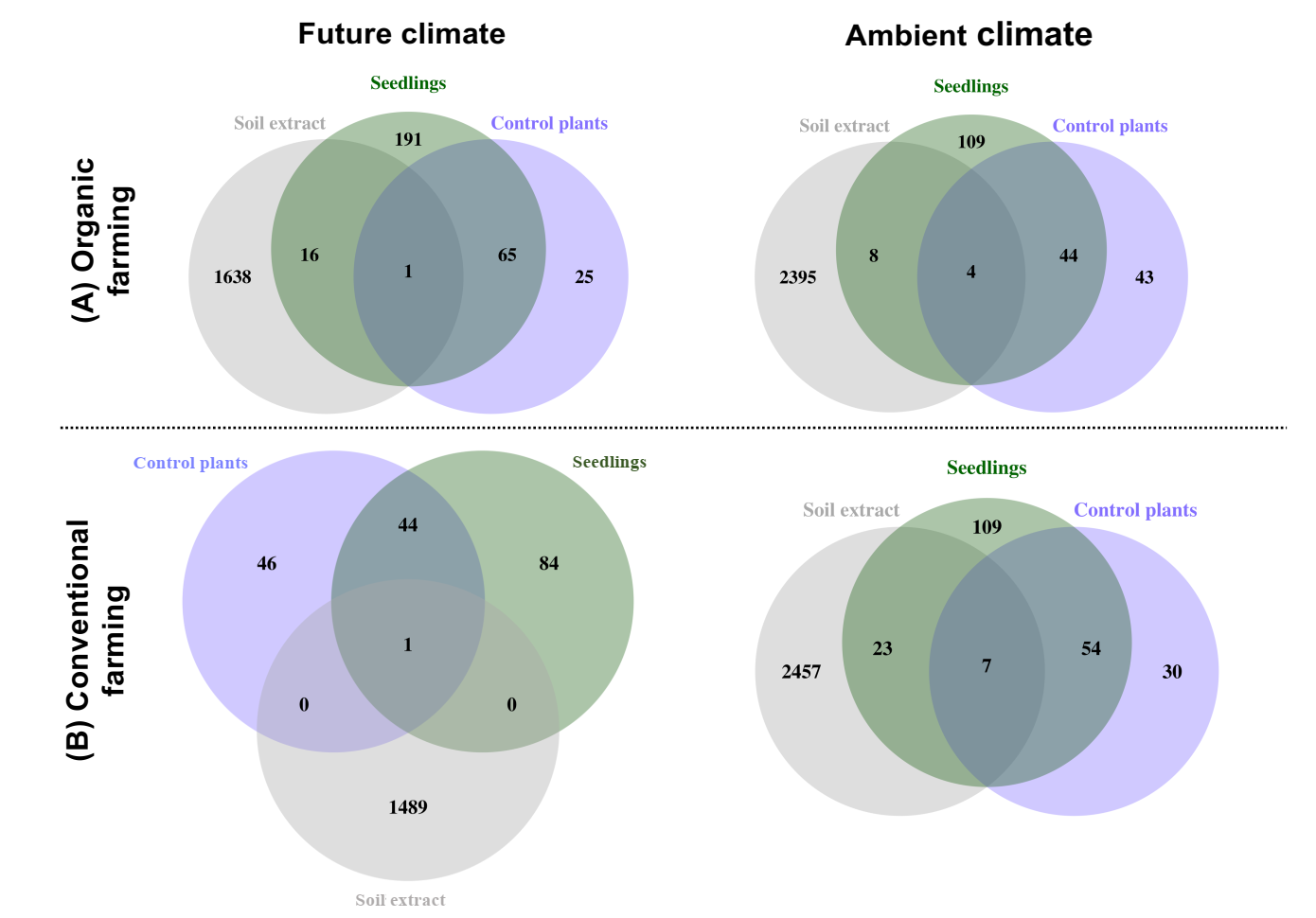


**Figure S2.** The number of shared and unique bacterial ASVs between the bacterial communities of inoculated seedlings and their respective inoculum (soil extracts) to the number of shared and unique ASVs between the communities of non-inoculated control plants and the same inoculum. The pattern is shown for the plant in which the seed was inoculated with a microbial extract from (A) organic and (B) conventional farming under future and ambient climates separately.


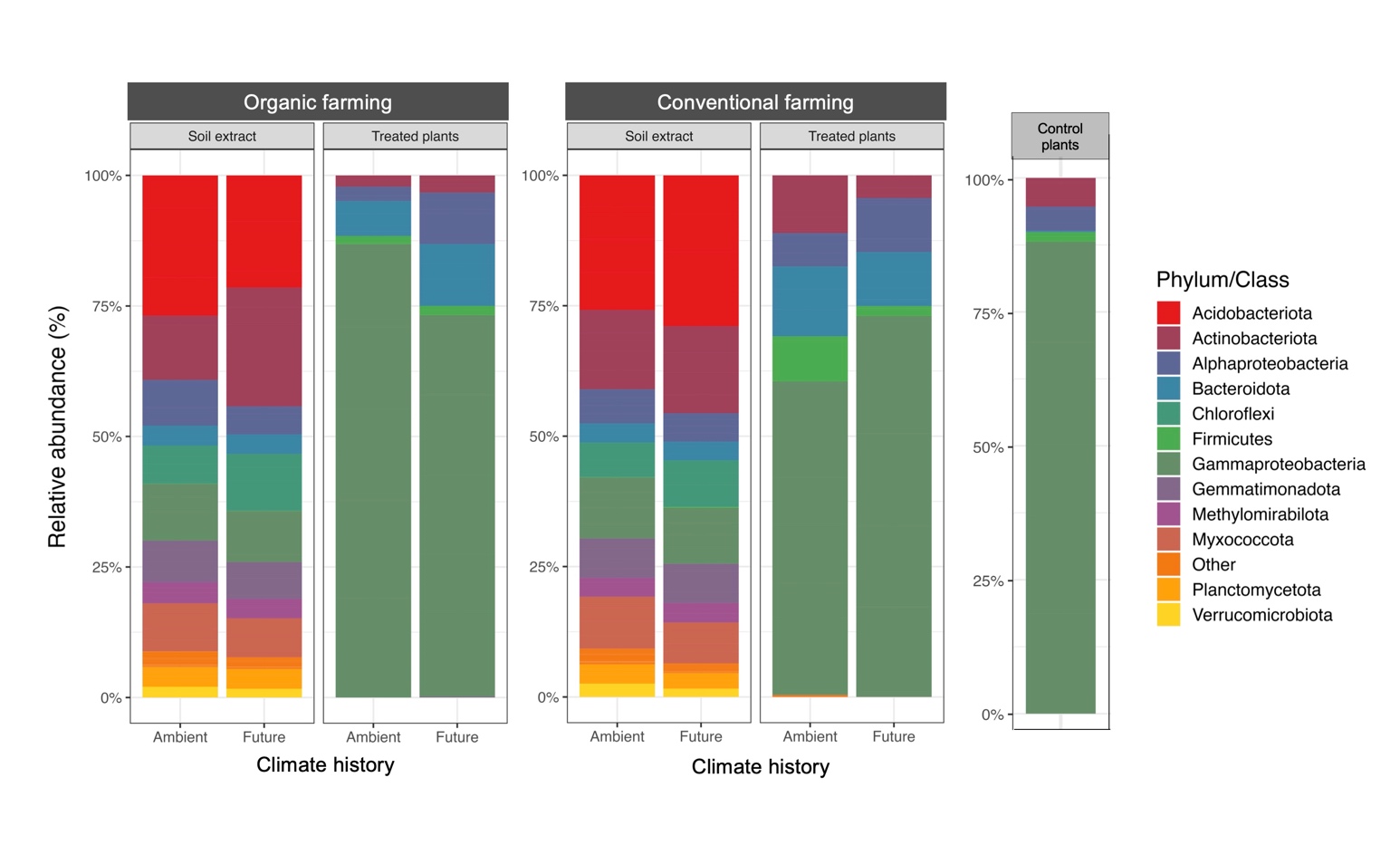


**Figure S3.** Relative abundance of the most abundant bacterial phyla (at the classes level for *Proteobacteria*) associated with soil extracts, non-inoculated (control), and inoculated (treated) plants. The relative abundance is shown according to inoculated plants and their corresponding soil microbial extracts with different farming (organic vs. conventional) and climate (future vs. ambient) histories.


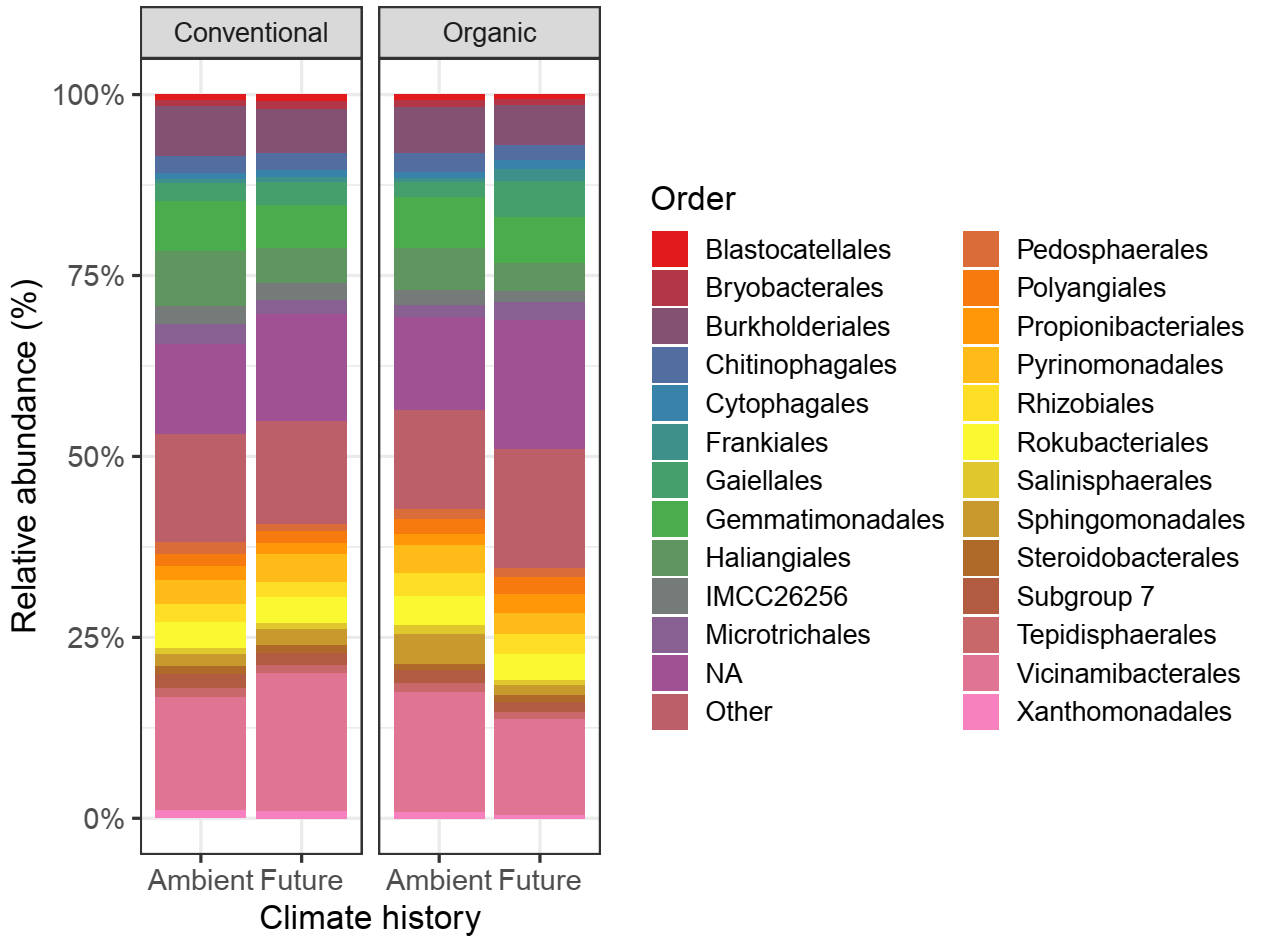


**Figure S4.** Relative abundance of the most abundant bacterial order associated with soil microbial extracts. The relative abundance is shown according to microbes extracted from different farming (organic vs. conventional) and climate (future vs. ambient) histories.


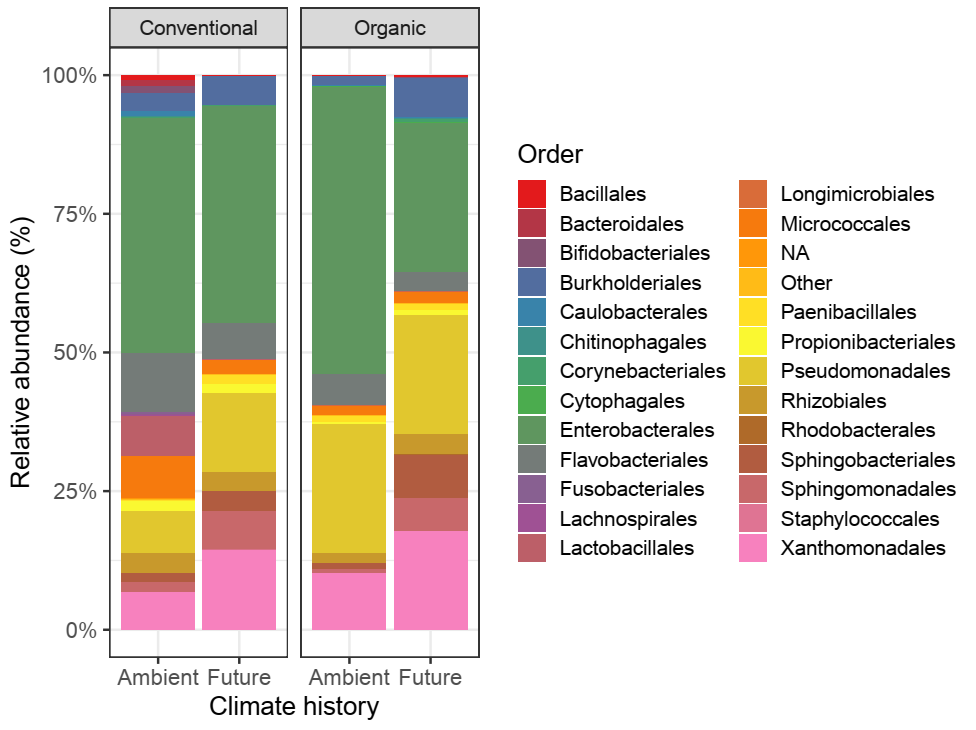


**Figure S5.** Relative abundance of the most abundant bacterial order associated with inoculated (treated) plants. The relative abundance is shown according to inoculated plants based on soil microbial extract from different farming (organic vs. conventional) and climate (future vs. ambient) histories.
